# Clinicians’ tolerance for uncertainty and communication about uncertainty with older adults –a standardized patient assessment study

**DOI:** 10.1186/s12877-025-06705-y

**Published:** 2025-12-08

**Authors:** Marij Hillen, Charlotte Nijskens, Sharon Wezeman, Remco Franssen, Ellen Smets

**Affiliations:** 1 Department of Medical Psychology, Amsterdam UMC, University of Amsterdam, P.O. Box 22660, Amsterdam, 1100DD The Netherlands; 2 Quality of Care, Amsterdam Public Health, Amsterdam, The Netherlands; 3 Department of Internal Medicine, Amsterdam UMC, University of Amsterdam, Amsterdam, The Netherlands

**Keywords:** Uncertainty, Patient-provider interaction, Physician-patient relation, Geriatrics, Communication, Observational coding

## Abstract

**Background:**

Uncertainty is omnipresent in care for older adults. Resident-physicians may be challenged to manage and communicate about such uncertainty. We aimed to 1) describe how resident-physicians discussed uncertainty with an older adult patient and involved them in decisions; and 2) assess how resident-physicians’ tolerance for uncertainty predicted communication about uncertainty and efforts towards patient involvement.

**Methods:**

A video-recorded, standardized patient assessment study was conducted involving resident-physicians in internal medicine. Participants conducted online consultations with a standardized geriatric patient. Self-report questionnaires assessed participants’ tolerance for uncertainty, feelings of ambivalence, and satisfaction with communication. Systematic observational coding assessed how residents communicated about uncertainty and involved the patient in decision-making. Descriptive statistics and correlations between observed communication and self-report measures were tested.

**Results:**

Residents (*N* = 37) expressed uncertainty on average 14 times per consultation (range 7–24), most often implicitly (67%). They primarily used information focused approaches (72%, e.g., explaining uncertainty). Emotion-focused approaches were scarcer (21%, e.g., facilitating patients’ coping with uncertainty). Tolerance for uncertainty was relatively high, and unrelated to residents’ uncertainty communication. Residents involved the patient in decision making to an acceptable degree. Patient involvement was not associated with residents’ uncertainty tolerance. Consultations with higher patient involvement included more frequent uncertainty expressions by residents (r_p_=0.38, *p* = .02).

**Conclusions:**

More solid evidence is needed on the relation between residents’ uncertainty tolerance and communication within care for older adult patients. Research evidence could guide reflective education to support residents in managing uncertainty, as well as skills training to enhance uncertainty communication.

**Supplementary Information:**

The online version contains supplementary material available at 10.1186/s12877-025-06705-y.

## Introduction

Medical practice is steeped in uncertainty relating to diagnosis, prognosis as well as treatment [1]. Uncertainty is particularly salient in the care for older adults- a population cared for not only by geriatricians but by physicians in most medical specialties [2, 3]. First, many older adult patients suffer from multi-morbidity. The complex interplay between various conditions, combined with wide variation in individual patients’ health, induces particularly high levels of uncertainty to which guidelines do not always provide answers [4, 5]. Moreover, the vulnerability associated with multi-morbidity at the end of life raises moral uncertainty about whether and which treatments will be in the patient’s best interest. Second, specific prognostication of older adults’ life expectancy is challenging for clinicians [6, 7]. Third, due to cognitive impairment, clinicians frequently need to communicate with third parties (e.g., partner, children). This may create uncertainty at multiple levels: a lack of insight into the patient’s preferences, and moral uncertainty about how to deal with informed consent and patient autonomy in decision making [8, 9]. Finally, scientific evidence about the effectiveness of medical treatment is limited and based mainly on younger patients without comorbidities. This lack of specific evidence leaves uncertainty about the (side) effects of treatment and prognosis [4, 10]. Thus, the care for older adults is steeped with uncertainty.

Clinicians require a strong ability to manage and tolerate uncertainty, i.e., ‘tolerance for uncertainty’. Tolerance for uncertainty has been defined as an individual’s various positive and negative responses to their meta-cognitive awareness of ignorance [11, 12]. Such responses to uncertainty may occur at a cognitive level (e.g., acknowledging vs. denying the presence of uncertainty), emotional level (e.g., experiencing anxiety vs. hope in the face of uncertainty), and behavior level (e.g., avoiding decisions vs. seeking resolution). Initial research suggests that clinicians who are more tolerant of uncertainty experience less difficulty in performing their work, and experience less stress and fear of making medical mistakes [12, 13]. A higher tolerance for uncertainty has moreover been associated with lower levels of burnout among students and physicians [14]. Finally, clinicians with a lower tolerance for uncertainty have self-reported higher rates of test-ordering [13]. In summary, clinicians’ tolerance for uncertainty appears crucial for their own functioning and for their medical practice.

It is increasingly acknowledged that medical training needs to make deliberate efforts to strengthen clinicians’ tolerance for uncertainty [15, 16]. Yet, such education is still far from common practice [17]. There are indications that strong hierarchical structures prevent residents from learning to openly discuss uncertainty, teaching them instead to resolve uncertainty by consulting seniors [18, 19]. More focused training efforts may be needed to ensure residents obtain a richer variety in approaches to manage uncertainty, including ways to endure irreducible uncertainty [20]. Such skills and insights could benefit residents’ professional and personal well-being.

Strengthening residents’ uncertainty management may additionally indirectly benefit patients. It has been suggested that clinicians’ tolerance for uncertainty is associated with their willingness to discuss uncertainty with patients. Particularly, preliminary evidence suggests that clinicians who are more comfortable in the face of uncertainty, are more willing and able to openly discuss uncertainty with patients [12, 13, 20]. This is important, as clinicians are nowadays expected to share uncertainty with patients: modern medicine entails patient engagement and involvement in decision making, both of which require that patients are openly informed about the uncertainties associated with their medical situation [21]. Yet, although the importance of openly discussing uncertainty with patients is widely acknowledged, physicians may currently be sub-optimally equipped to do so [13]. Moreover, presumed associations between uncertainty tolerance and communication about uncertainty have barely been researched.

Research addressing resident physicians’ communication of uncertainty so far has relied mostly on self-report, while direct interactional observations are largely lacking [22, 23].

We need insights in resident-physician’s approaches to discussing uncertainty with older adults, including their efforts to involve these patients in uncertainty regarding medical decision-making. Moreover, we need to better understand how residents’ uncertainty tolerance predicts their uncertainty communication. These insights can pave the way for interventions that better equip physicians in dealing with the uncertainties posed by current medical care for older adults. Eventually, this will enable patients to actively participate in conversations and decisions regarding their care, which ultimately benefits the quality of care.

We sought to (1) describe the ways in which resident-physicians discussed uncertainty when caring for older adult patients, and the degree to which they involved these patients in decisions. Moreover, we aimed to (2) assess how their tolerance for uncertainty predicted communication about uncertainty, and how it predicted the degree of patient involvement in decision-making.

## Methods

### Design

An observational, standardized patient assessment study was conducted in The Netherlands. This well-established method combines experimental control with an acceptable degree of ecological validity [24]. Resident-physicians conducted a medical consultation with a simulated older adult patient, and completed pre and post consultation questionnaires. The simulated patients’ behavior was kept constant across consultations using a script and specific instructions. Resident-physicians’ communication behavior was observationally coded. The study was exempted from the Medical Research Regulations Involving Human Subjects Act by the Medical Ethics Committee of Amsterdam University Medical Centers, location AMC (W20_425 # 20.472).

### Setting and participants

Participants were Dutch resident-physicians in Internal Medicine (hereafter referred to as ‘residents’), recruited through convenience sampling. Each participant conducted a standardized consultation with the same white, male, highly experienced actor between October 2020 and November 2021. The standardized consultation was a preparatory assignment prior to a one-day training course focused on uncertainty in geriatric practice. The actor’s performance quality and consistency was ensured using: (a) elaborate instructions on the portrayed patient’s background, characteristics and behavior, as well as on specific questions and behaviors to be displayed; (b) a pilot consultation with a research assistant acting as clinician, where one of the authors (MH) provided feedback on realistic play; and (c) three items assessing realism included in the resident questionnaire (see ‘Procedure and measurements’). Consultations were online, because of Covid-19 restrictions, and were video-recorded. Participants provided informed consent and were informed that the recorded consultation would be used for research focusing on medical communication.

### Procedure and measurements

**Fig. 1:**
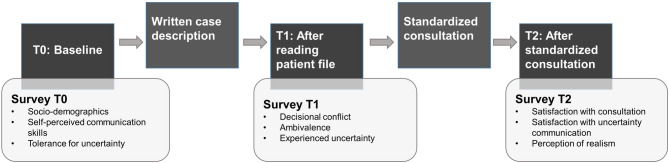
Study procedures

Residents, the simulated patient, and a research assistant logged onto a secure digital meeting (GoToMeeting, Citrix Online LLC, Goleta, USA, 2013). Residents completed an online survey (T0) and subsequently read the patient case description for the simulated scenario. The scenario involved an 80-year-old male white multi-morbid patient with an anemia, potentially caused by a malignant colon tumor. Detecting a tumor would involve invasive testing (see Table 1 for full scenario). After reading the scenario, residents completed a second survey (T1). Next, they conducted the standardized consultation with a simulated older adult patient, and their consultation was video recorded using the meeting software. Finally, residents completed a third survey (T2; see Fig. 1).

**Table 1:** Scenario

• The scenario described an 80-year-old patient with mild cognitive impairment and multi-morbidity, who was referred to the outpatient clinic for evaluation of an iron deficiency anemia. One of the possible causes of an iron deficiency anemia is a malignant colon tumor. Detecting a possible tumor would require invasive diagnostic testing including a colonoscopy. • The case involved multiple sources of uncertainty, including (1) various potential causes of the patient’s anemia, ranging in severity; (2) uncertainty about the patient’s physical ability to undergo invasive diagnostic testing; (3) doubt whether a possible tumor, if detected, could or should still be treated due to the patient’s poor physical condition; (4) doubt about the patient’s level of cognitive function and the ability to understand the medical information and considerations; and (5) unpredictability about the effects of iron supplementation on the anemia and patient’s symptoms. • Residents were instructed to consult with the patient about the decisions whether to pursue diagnostic testing and/or start iron supplementation.	

All self-reported measures are reported in detail in Table 2. In summary, at baseline (T0), we assessed residents’ *background characteristics* and their professional and personal *Tolerance for uncertainty*, using the Physicians’ Responses to Uncertainty Scale (PRUS; Cronbach’s α in the current study = 0.80) and the Intolerance for Uncertainty Scale (IUS-12; α = 0.82), respectively [25, 26]. At T1, residents reported their *ambivalence* about the case description, using the Subjective Ambivalence Questionnaire [27]. At T2, residents reported *Satisfaction about their own communication during the consultation* (modified Patient Satisfaction Questionnaire (PSQ; α = 0.85) [28] and their *Perception of realism* [29].

**Table 2:** Overview of self-reported study measures

Time-point	Construct	Instrument	#items, dimensions, response scale	Example item	Internal consistency (α)
T0	Background characteristics	Separate items	4 items (age; gender; years of residency training; total number of hours having received communication training throughout medical training)		N/A
	Tolerance for uncertainty - professional	Physicians’ Reactions to Uncertainty Scale (PRUS; Gerrity et al., 1990)	15 items; 4 dimensions (Anxiety due to uncertainty, Concern about bad outcomes, Reluctance to disclose uncertainty to patients, Reluctance to disclose mistakes to physicians); 6-point Likert scale (1 = Strongly disagree – 6 = Strongly agree)	‘I fear being held accountable for the limits of my knowledge’	0.80
	Tolerance for uncertainty - personal	Intolerance of Uncertainty scale (IUS; Carleton et al., 2007)	12 items; 5-point Likert scale (1 = Not at all typical of me – 5 = Very typical of me)	‘I always want to know what the future has in store for me’	0.82
T1	Ambivalence about the situation	Subjective Ambivalence Questionnaire (Priester & Petty, 1997); adapted for current study content.	3 items referring to the desirability of diagnostic testing (colonoscopy); 0–100 visual analogue scale	‘With regards to this topic, I have conflicting feelings’	N/A
T2	Satisfaction about own communication during the consultation	Patient Satisfaction Questionnaire (PSQ; Zandbelt et al., 2004) - slightly adapted to apply to the current situation	5 items; 0–100 visual analogue scale (0 = not at all to 100 = very much)	‘How satisfied are you about the information you provided to the patient?’	0.85
	Perception of realism	Previously developed items (Medendorp et al., 2020)	5 items; 7-point Likert scale (1 = not at all to 7 = very much)		N/A

Video-recorded online consultations were observer-coded using Noldus ‘The Observer’ software [30]. First, recordings were coded for residents’ communication about uncertainty using a specific coding instrument developed based on previous work, i.e., a literature review on uncertainty communication [31], a qualitative analysis of uncertainty communication in oncology [32], and extensive research team discussions. The coding instrument was initially developed within a Parkinson’s disease context (*Kurpershoek et al.*,* submitted*). For the current study, adaptations were made to align the coding instrument with the current geriatric setting (e.g., removing irrelevant/adding relevant topics of uncertainty). A highly specific coding manual was created (see Appendix A). Uncertainty expressions were defined as ‘any utterance in which the resident-physician explicitly or implicitly conveyed an awareness of not knowing something’. For each identified expression of uncertainty by the physician, we coded: [1] to which of 12 pre-defined topics the uncertainty pertained [2], whether uncertainty was expressed explicitly (e.g., ‘I don’t know’) or implicitly (e.g., ‘it is possible that…’), and [3] which of 14 pre-defined communicative approach(es) the physician used in conveying uncertainty (see Appendix A). All consultations were independently coded by at least two out of three raters (SW, SG, MH). Inter-rater reliability was assessed using Kappa in two ways: taking into account only main codes (occurrence and timing of the expressions and topic), and including all sub-codes. The tolerance window was set at 3 s. Kappa after coding two consultations was slight (mean κ = 0.26 (main codes) and mean κ = 0.20 (all sub-codes)) [33]. After three more consultations, Kappa was still low insufficient and variable (mean κ = 0.29 (main codes) and mean κ = 0.20 (all sub-codes)). We therefore continued double-coding and holding consensus discussions for every remaining consultation, resulting in slightly higher but still suboptimal Kappas over the remaining 32 consultations (mean κ = 0.47 (main codes) and mean κ = 0.30 (all sub-codes)).

Second, we coded consultations for the degree to which clinicians involved the patient in decision-making, using the 12-item validated ‘Observing patient involvement in decision making’ scale (OPTION-12; see Table 1) [34]. The detailed OPTION-12 coding manual was slightly adjusted to the specific context and situation of our study, and can be requested from the authors. Twelve items were scored on a 5-point Likert scale (0 = behavior is not observed to 4 = behavior is observed and executed to a high standard). According to scoring instructions, sum scores were converted to a 0 to 100 scale to facilitate interpretation. Two researchers (RR and MH) independently coded consultations and held regular consensus meetings after each 1 or 2 coded consultations. Where necessary, further detail and modifications were made to the coding manual. Once sufficient inter-rater reliability was established after 11 consultations, one researcher (RR) single coded the remaining consultations. Average inter-rater reliability for the 11 double-coded consultations was κ = 0.62 (range = 0.45–0.87.45.87), indicating substantial agreement [33]. Internal consistency was high at α = 0.81.

### Analysis

Twelve pre-defined topics of uncertainty were divided into *4 main topics* (i.e., causes of anemia, diagnostic testing, treatment options after testing, and iron supplementation; see Table 1). Of in total *14 communication approaches*, 8 were categorized as *emotion-focused* (e.g., exploring the patient’s uncertainty responses) and 6 as *information-focused* (e.g., outlining potential scenarios and consequences). Descriptive statistics (i.e., frequencies, percentages, means, SDs, ranges) were calculated to describe the sample, for self-report measures (PRUS, IUS-12, ambivalence, PSQ, realism), and for coded communication data (frequency, explicitness, topics, communicative approaches of uncertainty expressions, and OPTION-12 scores).

Using Pearson’s correlations, we tested relations between observed communication and self-report measures. We tested associations between physicians’ self-reported tolerance for uncertainty (PRUS and IUS-12 separately) and their: (a) self-reported ambivalence (T1); (b) observed uncertainty communication behavior (i.e., frequency, topic, explicitness, communicative approaches of uncertainty expressions); (c) degree of involving the patient in decision making (OPTION-12); and (d) self-reported satisfaction (PSQ).

## Results

### Sample characteristics

Table 3 shows characteristics of the 37 participating residents. Median years passed since the start of residency was 2 (range 0–7). Residents rated their own communication skills on average with 66.11 (SD = 13.36, range 30–85, potential range 0–100). Average video consultation duration was 17 min (range: 9–29).

**Table 3:** Sample characteristics of included resident physicians (N=37)

	Mean (SD), range
**Age**	32.19 (2.13), 28–37 years
**Years since start of residency (n = 29)**	2.04 (1.38), 0–7
**Communication training in undergraduate medicine ** *(self-estimated*,* # hours)*	33.87 (19.06), 10–80
**Communication training in residency program ** *(self-estimated*,* # hours)*	12.60 (19.45), 0–100
	***n (%)***
**Identified gender**	
Female	18 (51%)
Male	19 (49%)
**Field of specialty**	
Cardiology	6 (16%)
Geriatrics	5 (14%)
Hematology	5 (14%)
Infectious disease	5 (14%)
Oncology	4 (11%)
Pulmonology	3 (8%)
Endocrinology	2 (5%)
Other	4 (11%)
Not yet decided	3 (8%)

### Descriptive characteristics of questionnaire variables

Mean score for (personal) Intolerance for Uncertainty (IUS-12) was 2.06 (SD = 0.50, range 1.33–3.67, potential range 1–5), and for (professional) Reactions to uncertainty (PRUS) was 2.71 (SD = 0.53, range 1.53–3.60, potential range 1–6). Mean ambivalence score was 51/100 (SD = 19, range 10–94). Residents’ mean retrospective satisfaction with communication (PSQ) was 48/100 (SD = 13, range 14–68). Mean perceived realism was 5.70/7.00 (SD = 0.79, range 3.67–7.00.67.00).

### Uncertainty communication and decisional approach

Descriptive data for uncertainty expressions are provided in Table 4. Residents made on average 14.03 uncertainty expressions per consultation (SD = 3.93, range 7 to 24). Of these, one third (33%) were explicit and two thirds (67%) implicit. Uncertainty expressions pertained mostly to the cause of the patient’s anaemia and symptoms (53%). Table 5 displays some salient examples of uncertainty expressions for each communication approach. Residents most often (72%) used information-focused approaches when expressing uncertainty, and the most frequent approach was *outlining a follow-up plan to reduce uncertainty* (22% of all uncertainty expressions). Emotion-focused approaches amounted to 21% of residents’ expressions, for example by warning for ongoing uncertainty or offering a sense of hope. For 7% of uncertainty expressions, no specific communication approach was observed.

**Table 4:** Observed characteristics of clinicians’ uncertainty expressions

	M (SD), range		Percentage
**Number of uncertainty expressions** Explicit Implicit	**14.03 (3.93)**, **7–24** 4.54 (2.16), 1–12 9.49 (3.35), 3–18	- 33% (16), 8–70 67% (14), 30–92	
**Topics of uncertainty**			
Causes of anemia (*potential causes of anemia and blood loss*,* explanations for symptoms*)	7.41 (2.18), 3–12		53% (9), 33–73
Diagnostic testing (*diagnostic test options*,* associated risks and desirability*)	2.76 (1.79), 0–9		20% (13), 0–60
Treatment options after diagnostic testing (*potential treatment options*,* associated risks and desirability*)	2.24 (2.14), 0–9		16% (13), 0–45
Supplementary iron therapy (*effects on symptoms and on anemia*,* side effects*)	2.92 (1.40), 0–5		14% (11), 0–44
**Communicative approaches when expressing uncertainty**			
***No specific approach observed***	***0.97 (0.90)***,*** 0–3***		***7% (6)***,*** 0–27***
***Emotion-focused approaches***	***2.89 (1.88)***,*** 0–8***		***21% (14)***,*** 0–70***
Invite patient perspective/preference	0.86 (0.92), 0–3		
Temper hope	0.81 (1.15), 0–6		
Offer hope	0.51 (0.61), 0–2		
Caution for prolonged uncertainty	0.35 (0.68), 0–3		
Explore patient’s uncertainty responses	0.27 (0.61), 0–2		
Discuss potential ways of coping with uncertainty	0.03 (0.16), 0–1		
Provide emotional support or facilitate emotions	0.03 (0.16), 0–1		
Emphasize continued involvement	0.03 (0.16), 0–1		
***Information-focused approaches***	***10.16 (3.32)***,*** 3–17***		***72% (13)***,*** 30–100***
Normalize one’s own uncertainty	2.70 (1.65), 0–6		
Offer a follow-up plan by outlining next steps	2.32 (1.20), 0–5		
Outline potential scenarios and consequences	2.32 (1.67), 0–7		
Counterbalance uncertainty with certain aspects	1.95 (1.22), 0–5		
Explain reason for uncertainty	0.84 (1.01), 0–4		
Explain uncertainty understandably	0.03 (0.16), 0–1

**Table 5:** Examples of observed communicative approaches to expressing uncertainty

**Emotion-focused approaches **	**Example**
Invite patient perspective/preference	Do you have an idea of what you would prefer? Would you prefer to try out treatment for a stomach ulcer, or an investigation of the colon?
Temper hope	Chances are high that you will not see huge effects of the iron tablets, but it may help a little.
Offer hope	Thankfully, taking iron tablets can often strongly improve symptoms like fatigue.
Caution for prolonged uncertainty	If we decide not to do the investigation [colonoscopy], we cannot make a diagnosis of colon cancer. That can create uncertainty for you […] that can be unpleasant.
Explore patient’s uncertainty responses	Do you think you can live with the uncertainty, or do you necessarily want to know what is or is not present in your colon?
Discuss potential ways of coping	What would help you deal with that uncertainty about the diagnosis? Would it maybe help to discuss this with your close ones?
Provide emotional support or facilitate emotions	I wish I could give you that certainty right away.
Emphasize continued involvement	It is very difficult to predict how it [a potential cancer] would develop. You could for example get obstipation. But I would discuss that with the GP to make sure you would get adequate support.
**Information-focused approaches**	
Normalize one’s own uncertainty	Possibly your iron deficiency is due to colon cancer, but I can impossibly know that without any further tests.
Offer a follow-up plan by outlining next steps	We think you are losing blood somewhere, but why is that? To find out, we could do an extra test to look at certain bacteria in the stomach.
Outline potential scenarios and consequences	That iron deficiency can have various reasons. For example, that could be a stomach bacteria. If that is the case, we can give you antibiotics. But it could also result from a process … a tumor in the colon.
Counterbalance uncertainty with certain aspects	In your situation we don’t know what the precise cause of the iron deficiency is. But what *is *certain is that you have a deficiency.
Explain reason for uncertainty	I don’t know if it is the right thing to subject you to a very burdensome investigation, that can have many side effects. To eventually conclude that you are not fit for the surgery. I don’t know if that would be good advice.
Explain uncertainty understandably	Well, if it is cancer and we don’t treat it, then it is something that you will very likely eventually die from.

Mean OPTION12 raw score was 25.38/48.00 (SD = 7.17; range 9–37), and when converted to a 0–100 scale was 53.10 (SD = 15.19; range 19–77). Mean scores on individual items ranged from 0.30 for item 3 (‘*The clinician assesses the patient’s preferred approach to receiving information to assist decision making*’) to 2.95 for item 12 (‘*The clinician indicates the need to review the decision (or deferment)*’; see Appendix B).

### Correlations with tolerance of uncertainty

Residents’ general tolerance of uncertainty (IUS-12) did not predict *ambivalence* (r_p_=−0.02, *p* =.99). Lower professional tolerance of uncertainty (PRUS) was related to somewhat higher levels of experienced ambivalence (r_p_=0.35, *p* =.04), indicating that residents with more negative reactions to professional uncertainty, felt more ambiguous about the clinical situation. Regarding *uncertainty communication*, neither IUS-12 nor PRUS scores were associated with residents’ frequency or explicitness of uncertainty expressions, nor with their rate of using information-focused or emotion-focused communication approaches (all r_p_ *n.s.*). Separate testing of the relation between tolerance of uncertainty (IUS-12 and PRUS) and the most frequently observed communication approach (outlining a follow-up plan), showed no significant association for IUS-12, but a positive correlation for PRUS (r_p_=0.33, *p* <.05). The latter finding suggests that residents with more negative reactions to professional uncertainty were more likely to provide patients with a concrete action plan to reduce uncertainty. Tolerance of uncertainty (either IUS-12 or PRUS) was not related to residents’ *satisfaction with their own communication* (PSQ; *n.s.*).

Regarding involving *patients in decision making*, we found no associations between residents’ tolerance of uncertainty (IUS-12 or PRUS) and OPTION-12 scores (r_p_=0.22, *p* =.18 and r_p_=0.03, *p* =.89, respectively). Thus, residents with higher tolerance to uncertainty were not more likely to involve patients in decision making. Frequency of uncertainty expressions was positively associated with OPTION-12 scores (r_p_=0.38, *p* =.02), i.e., residents who expressed uncertainty more frequently, more actively involved patients in decision making.

When exploring correlations between residents’ age, gender and years of residency training, we found no associations, except between age and explicit/implicit uncertainty communication (r_p_=0.33): older residents were more likely to express uncertainty implicitly.

## Discussion

We investigated resident-physicians’ ways of managing and discussing uncertainty in a highly uncertain geriatric context. In simulated patient consultations, resident-physicians were found to express uncertainty mostly implicitly, using information-focused communication approaches such as explaining the reason for uncertainty. Residents’ tolerance for uncertainty was relatively high and mostly unrelated to their uncertainty communication, although residents with lower professional uncertainty tolerance more frequently provided a follow-up plan to reduce uncertainty. Residents involved patients in decision making independent of their uncertainty tolerance, and to a higher degree compared to clinicians in previous observational research [35]. Those residents who involved patients more, also expressed uncertainty more frequently.

Our findings that residents expressed uncertainty frequently, yet mostly implicitly, aligns with observations from primary care, in which explicit expressions of uncertainty were more scarce than implicit uncertainty expressions [36]. Such implicit expressions may be used to avoid openly acknowledging a lack of medical knowledge, or to mitigate unpleasant uncertainty in the patient [37]. Indeed, some previous research suggests that patients are more satisfied with indirect or implicit expressions of uncertainty compared to clear uncertainty displays, such as consulting a book [38, 39]. However, this research dates back more than 20 years, and due to increased patient participation and autonomy in recent years, patients may nowadays respond more favorably to clinicians’ explicit expressions of uncertainty.

Residents in our study were observed to use primarily information-focused approaches to discuss uncertainty. This aligns with previous research in clinical practice [40]. Such information-focused approaches may have important value: for example, when the clinician explains why something cannot be known, this may enhance patient understanding. Yet, these approaches also have their limitations. Specifically, we frequently observed residents outlining a plan forward to reduce uncertainty – particularly residents with a lower uncertainty tolerance. This suggests that particularly residents with lower tolerance may have felt more uncomfortable with uncertainty and wanted to eliminate their discomfort by envisioning how the uncertainty could be reduced [20]. Since uncertainty often cannot be (fully) eliminated, particularly in care for older adults, both residents and patients may benefit from more emotion-focused guidance to manage inevitable uncertainty [31]. Previous research shows that in serious illness, affective communication by clinicians may help patients cope with their situation by decreasing uncertainty and anxiety and increasing self-efficacy and satisfaction [41]. Eventually, patients’ enhanced management and acceptance of uncertainty could reduce excessive needs for diagnostic testing – which can be invasive, expensive, and yield further uncertainty [42].

Our expectation that residents’ uncertainty tolerance would predict their communication about uncertainty with older adults was not confirmed. Clinicians’ uncertainty tolerance may in reality not be an important driver for how they discuss uncertainty with patients. Potentially, residents may have felt obligated to openly discuss uncertainty even when they felt uncomfortable about doing so. Alternatively, this finding may be due to our sample’s relatively high tolerance to uncertainty compared to studies within other medical contexts. This aligns with previous evidence, showing higher uncertainty tolerance among clinicians in internal medicine compared to other specialties [43, 44]. Relations between uncertainty tolerance and communication might be more pronounced in samples with more variable levels of uncertainty tolerance. Alternatively our measures to assess uncertainty tolerance may have been unable to detect existing associations between UT and uncertainty communication. Recently, uncertainty tolerance measures have been criticized for too simplistically operationalizing this personality characteristic [45]. In the traditional view of uncertainty tolerance, there is a strict distinction between ‘good’ and ‘bad’ ways to manage uncertainty. Recently, scholars have advocated for a more nuanced view of uncertainty tolerance, where both tolerance and intolerance may have beneficial aspects [45, 46]. Tolerance of uncertainty might, in these scholars’ eyes, need to be reframed as *‘comfort in the face of one’s subjective perception of one’s own ignorance about some aspect of a clinical situation*, i.e.,* comfort in the face of uncertainty’* [46]. Reframing uncertainty tolerance in this way, would require developing or modifying existing measures.

We found that residents involved older adult patients in medical decision making to a relatively high degree, compared to previous standardized and clinical studies using the same assessment instrument (OPTION-12) [35]. Potentially, these newly trained resident-physicians were more instilled with the necessity of active patient involvement compared to the relatively experienced clinicians observed in previous studies. Involving older adult patients in decision making about their own health is considered especially crucial, because their values and preferences can guide difficult trade-offs, for example, between maximizing quantity or quality of life [47, 48]. In The Netherlands, the importance of equipping clinicians for shared decision-making is now increasingly emphasized in both basic and continued medical education. which may have impacted resident communication [49, 50]. Alternatively, residents may have involved patients more because they knew their behaviors would be observed and were ‘at their best behavior’, being aware that shared decision making is nowadays considered best practice [51]. We additionally observed that in conversations with higher patient involvement, residents expressed more uncertainty. This finding reiterates that the routine implementation of SDM in clinical practice may cause patients to be more explicitly and frequently confronted with uncertainty regarding the risks, benefits and (lack of) evidence regarding treatment options. Considering the potentially adverse effects of uncertainty on patients, SDM training for residents should equip them with optimal ways to convey uncertainty and facilitate patients in managing it.

### Strengths and limitations

Through our standardized patient assessment design, we were able to balance ecological validity with some degree of experimental control. Validity of our design was supported by residents rating the consultations as realistic. Moreover, by standardizing the patient’s behavior, variations in clinician communication cannot be explained by patient variation. Yet, the lack of variation in the patient’s behavior simultaneously inherently limits this design. Also, even though residents’ assessments suggested they perceived the portrayed patient as realistic, the actor’s performance quality was not consistently monitored throughout data collection, which may have reduced validity. Moreover, standardized consultations were conducted online, due to Covid-19 restrictions. Although online consultations are increasingly implemented in medical care, residents’ communication may have differed from their offline communication as well as from their communication when not observed [52]. Another limitation that may have reduced generalizability is the absence of an informal carer in the simulated consultations. Although it was not uncommon during Covid-19 that patients visited their hospital appointment unaccompanied, it would be more common for older adults with mild cognitive impairment to bring an informal carer. Additionally, clinicians’ uncertainty management and communication in real clinical practice may differ from the simulated context in our current study. Clinicians’ behavior may have been impacted by the scenario which lacked the complexity of clinical reality, and by increased awareness of being observed – the so-called ‘Hawthorne effect’ [52]. Studies in real clinical practice are therefore needed to assess whether our findings are similar when patient characteristics and behavior are more variable, when consultations are conducted offline vs. online, and when patients are accompanied by informal caregivers.

Our systematic observational coding procedures enabled us to obtain real-time insights into communication about uncertainty as it unfolded, which is another strength. At the same time, our coding instrument, although developed based on prior work and extensively pilot-tested, still lacked thorough psychometric validation. We did not achieve sufficient inter-rater reliability scores to conduct single coding of recorded consultations. We addressed this by double-coding and discussing all consultations. While this procedure ensured reliability, more work is needed to establish the content validity of the coding instrument and its individual coding categories. Further development should ensure a coding instrument that yields good reliability while still capturing meaningful aspects of uncertainty communication. Finally, our sample was relatively small, due to practical restraints, which limited statistical power and generalizability. Future work on larger samples should further substantiate our findings. Future studies could additionally examine cultural variation across healthcare systems in how residents and other clinicians manage uncertainty, which may be influenced by factors such as fear of litigation and healthcare cost considerations.

### Implications for clinical practice

Our findings yield only tentative implications for medical training. Discussing uncertainty is an integral and inevitable part of medical care for older adult patients, and should be firmly embedded in (continued) medical education [46]. Integrating reflective practices in medical teaching can potentially facilitate students and clinicians to become more aware of their natural responses to uncertainty, and of the ways such uncertainty shapes their perceptions, emotions, and behavior [53–57]. For example, after being presented with a complex patient case, learners may be invited to discuss amongst each other their automatic affective and behavioral responses to the situation [56]. This may facilitate acceptance of inevitable uncertainty, as well as stimulate considering various approaches to managing uncertainty.

Clinicians could additionally be taught to use various approaches available to them to discuss uncertainty with older adult patients. Using information-focused ways (e.g., explaining why you can’t be certain, outlining potential scenarios and their implications) is useful and already common practice. Additionally, residents may learn to facilitate older patients in managing such uncertainty, to reduce potential negative consequences. Residents could be taught to facilitate patients’ uncertainty management by using more emotion focused approaches, for example, acknowledging the potential emotional impact of continued uncertainty, suggesting ways to cope with it, or emphasizing their own continued involvement [31]. Residents may also be taught to actively check with patients how they generally respond to uncertainty. Based on patients’ needs, clinicians could adjust their approach and level of detail in discussing uncertainty. Eventually, teaching residents optimal communication about uncertainty will not only benefit older adult patients’ well-being, but also enable them to constructively engage in decision-making.

## Conclusion

In this standardized patient assessment study, we found that resident-physicians expressed uncertainty to an older adult patient frequently, albeit mostly in an implicit and information-focused way. Residents’ uncertainty tolerance levels were high overall and mostly unrelated to their uncertainty communication. More solid evidence is needed on the impact of various approaches to discussing uncertainty on older adult patients, to guide clinicians in optimally conveying uncertainty and supporting patients in managing its impact. Resident training in geriatrics and beyond could include reflective teaching to facilitate uncertainty management, and might additionally teach residents various available approaches to uncertainty communication. Follow-up research of the relation between uncertainty tolerance and communication is needed in both naturalistic and more controlled settings.

## Supplementary Information

Supplementary Material 1.

Supplementary Material 2.

## Data Availability

Anonymized data and materials, excluding video recordings of standardized consultations (which cannot be fully anonymized), are available from the authors upon request.

## References

[1] Han PK, Klein WM, Arora NK. Varieties of uncertainty in health care: a conceptual taxonomy. Med Decis Making. 2011;31(6):828–38. 22067431 10.1177/0272989X11393976 PMC3146626

[2] van Iersel MB, Brantjes E, de Visser M, Looman N, Bazelmans E, van Asselt D. Tolerance of clinical uncertainty by geriatric residents: a qualitative study. Eur Geriatr Med. 2019;10:517–22. 34652805 10.1007/s41999-019-00199-9

[3] Loewenthal JV, Beltran CP, Atalay A, Schwartz AW, Ramani S. What’s Going to Happen? Internal Medicine Resident Experiences of Uncertainty in the Care of Older Adults. J Gen Intern Med. 2024;40:226–233. 10.1007/s11606-024-08720-y 10.1007/s11606-024-08720-y PMC11780066 38485878

[4] Boyd CM, Darer J, Boult C, Fried LP, Boult L, Wu AW. Clinical practice guidelines and quality of care for older patients with multiple comorbid diseases: implications for pay for performance. JAMA. 2005;294(6):716–24. 16091574 10.1001/jama.294.6.716

[5] Tinetti ME, Bogardus ST Jr, Agostini JV, Mass Medical Soc. Potential pitfalls of disease-specific guidelines for patients with multiple conditions. N Engl J Med. 2004. 10.1056/NEJMsb042458. 15625341 10.1056/NEJMsb042458

[6] Fried TR, Bradley EH, O’Leary J. Prognosis communication in serious illness: perceptions of older patients, caregivers, and clinicians. J Am Geriatr Soc. 2003;51(10):1398–403. 14511159 10.1046/j.1532-5415.2003.51457.x

[7] Thai JN, Walter LC, Eng C, Smith AK. Every patient is an individual: clinicians balance individual factors when discussing prognosis with diverse frail elderly adults. J Am Geriatr Soc. 2013;61(2):264–9. 23320808 10.1111/jgs.12098 PMC3573246

[8] Bramley L, Seymour J, Cox K, Samanta J. Perspectives on autonomy and advance decision-making: a qualitative study based on older people living with frailty and their carers. Med Law Int. 2024;24(1):54–81.

[9] Dalton AF, Golin CE, Esserman D, Pignone MP, Pathman DE, Lewis CL. Relationship between physicians’ uncertainty about clinical assessments and patient-centered recommendations for colorectal cancer screening in the elderly. Med Decis Making. 2015;35(4):458–66. 25712448 10.1177/0272989X15572828 PMC4424122

[10] Plsek PE, Greenhalgh T. The challenge of complexity in health care. BMJ. 2001;323(7313):625–8. 11557716 10.1136/bmj.323.7313.625 PMC1121189

[11] Hillen MA, Gutheil CM, Strout TD, Smets EM, Han PK. Tolerance of uncertainty: conceptual analysis, integrative model, and implications for healthcare. Soc Sci Med. 2017;180:62–75. 28324792 10.1016/j.socscimed.2017.03.024

[12] Strout TD, Hillen M, Gutheil C, Anderson E, Hutchinson R, Ward H, et al. Tolerance of uncertainty: a systematic review of health and healthcare-related outcomes. Patient Educ Couns. 2018;101(9):1518–37. 29655876 10.1016/j.pec.2018.03.030

[13] Alam R, Cheraghi-Sohi S, Panagioti M, Esmail A, Campbell S, Panagopoulou E. Managing diagnostic uncertainty in primary care: a systematic critical review. BMC Fam Pract. 2017;18:1–13. 28784088 10.1186/s12875-017-0650-0 PMC5545872

[14] Hancock J, Mattick K. Tolerance of ambiguity and psychological well-being in medical training: a systematic review. Med Educ. 2020;54(2):125–37. 31867801 10.1111/medu.14031 PMC7003828

[15] Geller G, Faden RR, Levine DM. Tolerance for ambiguity among medical students: implications for their selection, training and practice. Soc Sci Med. 1990;31(5):619–24. 2218644 10.1016/0277-9536(90)90098-d

[16] Geller G, Grbic D, Andolsek KM, Caulfield M, Roskovensky L. Tolerance for ambiguity among medical students: patterns of change during medical school and their implications for professional development. Acad Med. 2021;96(7):1036–42. 33149092 10.1097/ACM.0000000000003820

[17] Patel P, Hancock J, Rogers M, Pollard SR. Improving uncertainty tolerance in medical students: a scoping review. Med Educ. 2022. 10.1111/medu.14873 PMC9796811 35797009

[18] Farnan JM, Johnson JK, Meltzer DO, Humphrey HJ, Arora VM. Resident uncertainty in clinical decision making and impact on patient care: a qualitative study. Qual Saf Health Care. 2008;17(2):122–6. 18385406 10.1136/qshc.2007.023184

[19] Hamui-Sutton A, Vives-Varela T, Gutiérrez-Barreto S, Leenen I, Sánchez-Mendiola M. A typology of uncertainty derived from an analysis of critical incidents in medical residents: a mixed methods study. BMC Med Educ. 2015;15:1–11. 26537260 10.1186/s12909-015-0459-2 PMC4634904

[20] Han PK, Strout TD, Gutheil C, Germann C, King B, Ofstad E, et al. How physicians manage medical uncertainty: a qualitative study and conceptual taxonomy. Med Decis Making. 2021;41(3):275–91. 33588616 10.1177/0272989X21992340 PMC7985858

[21] Wet op de Geneeskundige Behandelingsovereenkomst (WGBO). [Dutch Medical Treatment Contracts Act] [Available from: https://www.knmg.nl/actueel/dossiers/patientenrechten/behandelingsovereenkomst-wgbo

[22] Cox CL, Miller BM, Kuhn I, Fritz Z. Diagnostic uncertainty in primary care: what is known about its communication, and what are the associated ethical issues? Fam Pract. 2021;38(5):654–68. 33907806 10.1093/fampra/cmab023 PMC8463813

[23] Hart J, Cox C, Kuhn I, Fritz Z. Communicating diagnostic uncertainty in the acute and emergency medical setting: A systematic review and ethical analysis of the empirical literature. Acute Med. 2021;20(3):204–18. 34679138

[24] Razavi D, Delvaux N, Marchal S, De Cock M, Farvacques C, Slachmuylder JL. Testing health care professionals’ communication skills: the usefulness of highly emotional standardized role-playing sessions with simulators. Psychooncology. 2000;9(4):293–302. 10960927 10.1002/1099-1611(200007/08)9:4<293::aid-pon461>3.0.co;2-j

[25] Gerrity MS, DeVellis RF, Earp JA. Physicians’ reactions to uncertainty in patient care. A new measure and new insights. Med Care. 1990;28(8):724–36. 2385142 10.1097/00005650-199008000-00005

[26] Carleton RN, Norton MA, Asmundson GJ. Fearing the unknown: a short version of the intolerance of uncertainty scale. J Anxiety Disord. 2007;21(1):105–17. 16647833 10.1016/j.janxdis.2006.03.014

[27] Priester JR, Petty RE. The gradual threshold model of ambivalence: relating the positive and negative bases of attitudes to subjective ambivalence. J Pers Soc Psychol. 1996;71(3):431. 8831157 10.1037//0022-3514.71.3.431

[28] Zandbelt LC, Smets EMA, Oort FJ, Godfried MH, de Haes JCJM. Satisfaction with the outpatient encounter. A comparison of patients’ and physicians’ views. J Gen Intern Med. 2004;19:1088–95. 15566437 10.1111/j.1525-1497.2004.30420.x PMC1494792

[29] Medendorp NM, Hillen MA, Van Maarschalkerweerd PE, Aalfs CM, Ausems MG, Verhoef S, et al. We don’t know for sure’: discussion of uncertainty concerning multigene panel testing during initial cancer genetic consultations. Fam Cancer. 2020;19(1):65–76. 31773425 10.1007/s10689-019-00154-4 PMC7026220

[30] Noldus LPJJ. The observer: a software system for collection and analysis of observational data. Behav Res Methods Instrum Comput. 1991;23(3):415–29.

[31] Medendorp NM, Stiggelbout AM, Aalfs CM, Han PK, Smets EM, Hillen MA. A scoping review of practice recommendations for clinicians’ communication of uncertainty. Health Expect. 2021;24(4):1025–43. 34101951 10.1111/hex.13255 PMC8369117

[32] van Someren JL, Lehmann V, Stouthard JM, Stiggelbout AM, Smets EM, Hillen MA. Oncologists’ communication about uncertain information in second opinion consultations: A focused qualitative analysis. Front Psychol. 2021;12:635422. 34135806 10.3389/fpsyg.2021.635422 PMC8201772

[33] Landis JR, Koch GG. The measurement of observer agreement for categorical data. Biometrics. 1977:159–74. 843571

[34] Elwyn G, Hutchings H, Edwards A, Rapport F, Wensing M, Cheung WY, et al. The OPTION scale: measuring the extent that clinicians involve patients in decision-making tasks. Health Expect. 2005;8(1):34–42. 15713169 10.1111/j.1369-7625.2004.00311.x PMC5060272

[35] Couët N, Desroches S, Robitaille H, Vaillancourt H, Leblanc A, Turcotte S, et al. Assessments of the extent to which health-care providers involve patients in decision making: a systematic review of studies using the OPTION instrument. Health Expect. 2015;18(4):542–61. 23451939 10.1111/hex.12054 PMC5060794

[36] Stortenbeker I, Houwen J, van Dulmen S, olde Hartman T, Das E. Quantifying implicit uncertainty in primary care consultations: a systematic comparison of communication about medically explained versus unexplained symptoms. Patient Educ Couns. 2019;102(12):2349–52. 31288956 10.1016/j.pec.2019.07.005

[37] Blanch DC, Hall JA, Roter DL, Frankel RM. Is it good to express uncertainty to a patient? Correlates and consequences for medical students in a standardized patient visit. Patient Educ Couns. 2009;76:300–6. 19604663 10.1016/j.pec.2009.06.002

[38] Ogden J, Fuks K, Gardner M, Johnson S, McLean M, Martin P, et al. Doctors expressions of uncertainty and patient confidence. Patient Educ Couns. 2002;48(2):171–6. 12401420 10.1016/s0738-3991(02)00020-4

[39] Johnson CG, Levenkron JC, Suchman AL, Manchester R. Does physician uncertainty affect patient satisfaction? J Gen Intern Med. 1988;3(2):144–9. 3357071 10.1007/BF02596120

[40] Prins S, Linn AJ, van Kaam AH, van de Loo M, van Woensel JB, van Heerde M, et al. How physicians discuss uncertainty with parents in intensive care units. Pediatrics. 2022;149(6):e2021055980. 35603505 10.1542/peds.2021-055980

[41] Van Vliet LM, Van der Wall E, Plum NM, Bensing JM. Explicit prognostic information and reassurance about nonabandonment when entering palliative breast cancer care: findings from a scripted video-vignette study. J Clin Oncol. 2013;31(26):3242–9. 23940230 10.1200/JCO.2012.45.5865

[42] Rogers WA, Walker MJ. Fragility, uncertainty, and healthcare. Theor Med Bioeth. 2016;37:71–83. 26906556 10.1007/s11017-016-9350-3

[43] Matteson MT, Smith SV. Selection of medical specialties: preferences versus choices. Acad Med. 1977;52(7):548–54. 10.1097/00001888-197707000-00002 874987

[44] Merrill J, Camacho Z, Laux L, Lorimor R, Thornby J, Vallbona C. Uncertainties and ambiguities: measuring how medical students cope. Med Educ. 1994;28(4):316–22. 7862004 10.1111/j.1365-2923.1994.tb02719.x

[45] Patel P, Hancock J. Uncertainty tolerance scales: weighing up the research. Med Educ. 2023. 10.1111/medu.15114 37157928

[46] Reis-Dennis S, Gerrity MS, Geller G. Tolerance for uncertainty and professional development: a normative analysis. J Gen Intern Med. 2021;36(8):2408–13. 33532966 10.1007/s11606-020-06538-y PMC7853704

[47] Shen MJ, Manna R, Banerjee SC, Nelson CJ, Alexander K, Alici Y, et al. Incorporating shared decision making into communication with older adults with cancer and their caregivers: development and evaluation of a geriatric shared decision-making communication skills training module. Patient Educ Couns. 2020;103(11):2328–34. 32475710 10.1016/j.pec.2020.04.032 PMC7572605

[48] Pel-Littel RE, Snaterse M, Teppich NM, Buurman BM, van Etten-Jamaludin FS, van Weert JC, et al. Barriers and facilitators for shared decision making in older patients with multiple chronic conditions: a systematic review. BMC Geriatr. 2021;21:1–14. 33549059 10.1186/s12877-021-02050-y PMC7866443

[49] Raamplan artsopleiding [Framework Medical Education]. Netherlands Federation of university medical centres; 2020.

[50] Handreiking & competentieset scholing medisch specialisten. De kunst Van Samen beslissen’ [Guideline & skills set for teaching medical specialists ‘the Art of shared decision Making’]. Utrecht, Netherlands: Federatie Medisch Specialisten [Federation Medical Specialists]; 2019.

[51] Salzburg statement on shared decision making. In: Salzburg GS, editor. Brit Med J (Clin res ed)2011. p. d1745. 10.1136/bmj.d1745 21427038

[52] Franke RH, Kaul JD. The Hawthorne experiments: first statistical interpretation. Am Sociol Rev. 1978:623–43.

[53] Ilgen JS, Watsjold BK, Regehr G. Is uncertainty tolerance an epiphenomenon? Med Educ. 2022. 10.1111/medu.14938. 36124815 10.1111/medu.14938

[54] Lazarus MD, Gouda-Vossos A, Ziebell A, Brand G. Fostering Uncertainty Tolerance in Anatomy Education: Lessons Learned from how Humanities, Arts and Social Science (HASS) Educators Develop Learners’ Uncertainty Tolerance. Anat Sci Educ. 2022. 10.1002/ase.2174 PMC10078696 35114066

[55] Stephens GC, Sarkar M, Lazarus MD. ‘I was uncertain, but I was acting on it’: A longitudinal qualitative study of medical students’ responses to uncertainty. Med Educ. 2023. 10.1111/medu.15269 37963570

[56] Scott A, Sudlow M, Shaw E, Fisher J. Medical education, simulation and uncertainty. Clin Teach. 2020;17(5):497–502. 31903672 10.1111/tct.13119

[57] Papanagnou D, Ankam N, Ebbott D, Ziring D. Towards a medical school curriculum for uncertainty in clinical practice. Med Educ Online. 2021;26(1):1972762. 34459363 10.1080/10872981.2021.1972762 PMC8409968

