# Supplementary material for: Clinicians’ tolerance for uncertainty and communication about uncertainty with older adults –a standardized patient assessment study
Source: BMC Geriatr. 2025 Dec 8;26:46. doi: 10.1186/s12877-025-06705-y (PMC12797895; doi:10.1186/s12877-025-06705-y)
Supplement: Supplementary file 1 — Supplementary Material 1. [file 12877_2025_6705_MOESM1_ESM.docx]

**Appendix A. Coding manual for clinicians’ uncertainty communication**

**within the context of a geriatric multi-morbid patient with anemia**

| General guidelines   - Codes are assigned:   - At the moment the uncertainty expression starts; or   - At the moment the sentence starts in which the uncertainty is expressed, if the beginning of the sentence is related tot hat uncertainty (e.g., ‘*And that’s where it becomes complicated, because we don’t know exactly how long that takes*’) - For each expression of uncertainty, code (see Figure 1):   - ***Subject****:* Whether the uncertainty is expressed by the patient or clinician;   - If expressed by the patient:     - ***Clinician response****:* clinician’s initial response to patient expression   - If expressed by the clinician:     - ***Topic****:* Topic to which the uncertainty pertains     - ***Initiative****:* Whether the uncertainty expression by the clinician spontaneously or in response to a patient cue     - ***Explicitness****:* Whether the uncertainty is expressed implicitly or explicitly     - ***Communication approach****:* which communication approach uses when expressing the uncertainty - Code only expression of uncertainty that are to some degree linked to the (diagnosis, cause, treatment, consequences of the) anemia. For example, uncertainty about the patient’s living situation is not coded. |
| --- |

Figure 1: visual display of the coding process

**SUBJECT**

*Specify the person who expresses the uncertainty*

- Patient **(P)** *🡪 Proceed to ‘Physician response’*
- Clinician **(A)** *🡪 Proceed to ‘Topic’*

**1. Physician response**

*Select the clinician’s initial, uninterrupted response to the uncertainty expression by the patient. Note: If the clinician’s response to a patient’s uncertainty expression constitutes an expression of uncertainty, code it first as a clinician response. Next, code it separately as a clinician expression of uncertainty, starting from the moment the clinician starts their expression.*

**(R-NE)** **Ignoring**

*The clinician does not respond to the expression, and/or proceeds with an unrelated topic.*

**(R-ER)** **Minimal acknowledgement**
*The clinician provides some form of acknowledgement of the uncertainty expression, such as ‘I see’, but nothing more.*

**(R-II)** **Indirect information provision**
*The clinician does not directly address the patient’s question or uncertainty, but starts providing other (related) information*

**(R-DI)** **Direct information provision**
*The clinician directly answers or addresses the patient’s expression of uncertainty or question.*

**(R-RE)** **Reflection**
*The clinician acknowledges, reflects on or confirms the patient’s experience of uncertainty.*

**(R-EX)** **Exploration**
*The clinician explores what the patient means exactly, or provides silence to invite the patient to elaborate.*

**2. TOPIC**

*Assign the most fitting topic to each expression of uncertainty made by the clinician. General information provision that does not pertain to the patient specifically should not be coded.*

**(B)** **Cause of symptoms**

**(B-VS) Explanation of symptoms**

*Degree to which the symptoms, such as fatigue, can be explained by anemia or another cause.*

**(B-GS) Consequences/risks of symptoms caused by anemia***How symptoms of anemia may affect the patient’s health.*

**(B-OB) Cause of anemia***What the underlying cause of the anemia could be, e.g. disease, inflammation, blood loss.*

**(B-OV) Cause of blood loss***What the cause of possible blood loss could be, e.g., stomach ulcer or colon cancer*

**(T)** **Diagnostic testing**

**(T-DT) Diagnostic test options**

*Which diagnostic tests are available and what would be the best choice*

**(T-WT) Desirability of diagnostic test**

*Desirability of diagnostic tests in terms of the burden they might place on patients, or*

*in terms of the balance between their risks/burden and what benefits they could yield.*

**(T-RT) Risks of diagnostic tests***How risky the diagnostic procedure itself or the preparation would be, considering the patient’s condition, or the risks of not conducting the diagnostic test.*

**(O) Surgery**

**(O-VB) Treatment options (after diagnostic testing)***Which treatment options (e.g., surgery) would be available after diagnostic testing reveals colorectal cancer.*

**(O-RO) Risk of surgery***The risk that surgery would entail for the patient.*

**(O-AD) Dependency of others as a result of surgery***To what extent the surgery (or its complications) would result in the patient being*

*dependent on external support in daily life.*

**(O-MA) Moral consideration about surgery***The balance between how the benefits of surgery would compare to the complications and risks. Note: if risks of surgery are only mentioned as part of a balance between pros and cons, code only as O-MA, not as O-RO.*

**(M) Treatment**

**(M-EI) Effects of iron suppletion on iron level**
*Whether and when the iron tablets will improve the patient’s iron.*

**(M-ES) Effects of iron suppletion on symptoms**
*Whether and to what extent the iron tablets will affect the patient’s experienced symptoms (e.g., fatigue).*

**(M-BW) Side effects of iron suppletion**

*The risk and nature of side effects due to iron suppletion.*

**(V)** **Other**

**(V-OV) Other topics**

*Topics that are not included in the coding scheme. This may include practical uncertainties, such as the duration until a test result becomes available. Note down in the comments what the specific topic is.*

**3. InitiatiVE***Specify for each uncertainty expression made by the clinician whose behavior caused the clinician to express uncertainty.*

**(I-IA) Clinician’s initiative**
*The clinician spontaneously expresses uncertainty, at their own initiative.*

**(I-IP) Patient’s initiative**
*The clinician expresses uncertainty prompted by an expression or question from the patient, i.e., at the patient’s initiative.*

**4. EXPLICITNESS**

*Specify for each uncertainty expression made by the clinician whether the uncertainty is explicitly or implicitly acknowledged.*

**(X-IM) Implicit**
*The uncertainty becomes apparent only from subtle words, without open acknowledgement of ‘not knowing’. Signal words are: possibly, maybe, in principle, we sometimes see, I think/suspect, it is possible that, we have to see if... Note that the presence of such signal words does not necessarily point to an uncertainty expression.*

**(X-EX) Explicit**
*The clinician openly mentions/acknowledges they don’t know something or something is unknown. Signal words are: difficult to say, I don’t know, honestly, we don’t know, you can’t know, it cannot be predicted. Note that the presence of such signal words does not necessarily point to an uncertainty expression.*

**5. COMMUNICATION APPROACH**

*For each uncertainty expression by the clinician, assign at least one and maximally two communication approaches from the list below, i.e., different ways used to convey uncertainty. Only if no specific approach is observed, assign ‘A-AL’. Multiple communication approaches may be visible for one uncertainty expression, but only assign two approaches if these can be observed independent of each other (e.g., ‘we simply cannot be sure (K-RO), but what we do know is… (K-OA)).*

**(A) No approach**

**(A-AL)** **No communication approach observed**
*The clinician simply expresses uncertainty, without using a specific communicative approach.*

**(K) Information focused**

**(K-RO) Providing a reason for uncertainty**
*The clinician explains to the patient what the underlying reason or cause of uncertainty is, and/or explains why we can’t know something. This includes normalizing ones own uncertainty.*

**(K-OA) Alternating uncertainty with certainty (counterbalancing)**
*After conveying the uncertainty, the clinician mentions what the known/certain aspects of the situation are, or provides the most likely explanation.*

**(K-SC) Outlining possible scenarios/explanations and consequences**
*The clinician discusses different potential future scenarios or explanations (e.g., test results, diagnoses, treatment options, treatment outcomes). In discussing prognosis, this may include the best, worst and most likely scenario/explanation. The clinician discusses the potential impact of each scenario on the patient’s life.*

**(K-OB) Explaining uncertainty in an understandable way**
*The clinician uses simple and clear language to explain uncertainty (e.g., risks/chances), for example by using an analogy or examples, or a clear structure. This entails more than simply mentioning the risk.*

**(K-CB) Checking the patient’s understanding of uncertainty**
*The clinician checks the patient’s understanding of the uncertainty they have conveyed.*

**(K-CB) Offering control by outlining future steps**
*The clinician presents the patient with a future plan with follow-up steps to reduce uncertainty, or outlines the most probable (future) scenario.*

**(E) Emotion focused**

**(E-WO) Warning for (continued) uncertainty**
*The clinician warns the patient that uncertainty will persist or that follow up steps may lead to renewed uncertainty.*

**(E-ET) Explorering the patient’s uncertainty tolerance**
*The clinician explores how the patient regards, manages and responds to uncertainty and/or to receiving uncertain information.*

**(E-CS) Generating coping strategies for uncertainty**
*The clinician helps the patient generate strategies to deal/cope with present and/or future uncertainty.*

**(E-HB) Offering hope**

*The clinician offers the patient hope, for example by alternating uncertain bad news with good news, or by emphasizing a positive expectation, e.g., the hope to find a diagnosis eventually.*

**(E-EB) Offering emotional support or space for emotional reaction**

*The clinician offers the patient emotional support with regards to the uncertainty (this may include silences), or acknowledges that the uncertainty can be emotionally straining. Note: only code if the support is specifically focused on the uncertainty.*

**(E-BB) Emphasizing availability**

*The clinician assures the patient that they (or other care team members) will be/remain available for the patient. Note: code only if the expression is linked to uncertainty.*

**(E-VP) Checking patient preference**

*The clinician actively involves the patient in a decision that involves uncertainty, so that the patient’s wishes/preferences can be considered.*

**(O) OTHER**

**(O-HT) Tempering hope**
*The clinician tries to temper the patient’s hopes, or manages (tones down) their expectations.*

**(O-OM)** **Steering patient perception/behavior by maximizing/minimizing uncertainty**

*The clinician observably emphasizes or minimizes the level of uncertainty, apparently to affect the patient’s perception or behavior (e.g., to soften a blow). Tempering hope is not included in this code.*

**(O-OS) Other approaches**

*Any observed approaches that are not included in the coding scheme. Note in the comments the specific approach observed.*
