# Supplementary material for: Clinicians’ tolerance for uncertainty and communication about uncertainty with older adults –a standardized patient assessment study
Source: BMC Geriatr. 2025 Dec 8;26:46. doi: 10.1186/s12877-025-06705-y (PMC12797895; doi:10.1186/s12877-025-06705-y)
Supplement: Supplementary file 2 — Supplementary Material 2. [file 12877_2025_6705_MOESM2_ESM.docx]

**Appendix B**. Descriptive statistics of OPTION-12 scores (N = 37)

| **Item Description** | | **Mean (SD)** | **Range** | **IRR (κ)*** | **Agreement (%)**** |
| --- | --- | --- | --- | --- | --- |
| **Total score (transformed, potential range 0-100)** | | 53.10 (15.19) | 19 - 77 | 0.62 | 67% |
| **1.** | The clinician draws attention to an identified problem as one that requires a decision making process. | 2.30 (1.24) | 0 - 4 | 0.87 | 91% |
| **2.** | The clinician *states* that there is more than one way to deal with the identified problem (‘equipoise’). | 2.24 (0.90) | 1 - 4 | 0.53 | 55% |
| **3.** | The clinician *assesses* the patient’s preferred approach to receiving information to assist decision making (e.g. discussion, reading printed material, assessing graphical data, using videotapes or other media). | 0.30 (0.57) | 0 - 2 | 0.48 | 73% |
| **4.** | The clinician *lists* ‘options’, which can include the choice of ‘no action’. | 2.49 (1.12) | 0 - 4 | 0.56 | 55% |
| **5.** | The clinician *explains* the pros and cons of options to the patient (taking ‘no action’ is an option). | 2.35 (0.82) | 1 - 4 | 0.78 | 82% |
| **6.** | The clinician explores the patient’s *expectations* (or ideas) about how the problem(s) are to be managed. | 1.86 (1.18) | 0 - 4 | 0.47 | 55% |
| **7.** | The clinician explores the patient’s *concerns* (fears) about how problem(s) are to be managed. | 2.51 (1.04) | 0 - 4 | 0.53 | 73% |
| **8.** | The clinician checks that the patient has *understood* the information. | 2.08 (1.26) | 1 - 4 | 0.84 | 80% |
| **9.** | The clinician offers the patient explicit *opportunities* to ask questions during the decision making process. | 2.49 (0.90) | 1 - 4 | 0.76 | 82% |
| **10.** | The clinician elicits the patient’s *preferred level of* *involvement* in decision-making. | 1.84 (0.99) | 0 - 3 | 0.45 | 45% |
| **11.** | The clinician indicates the need for a *decision* *making* (or *deferring*) stage. | 1.97 (1.12) | 0 - 4 | 0.57 | 64% |
| **12.** | The clinician indicates the need to review the decision (or *deferment)*. | 2.95 (1.27) | 0 - 4 | 0.57 | 55% |

Note: potential range for all individual items is 0-4. *IRR=inter-rater reliability (Cohen’s weighted Kappa). **Percentage of agreement
